# Comments on: A new screening of preterm birth in gestation with short cervix after pessary plus progesterone

**DOI:** 10.61622/rbgo/2025rbgo98

**Published:** 2025-11-18

**Authors:** Vittorio Unfer

**Affiliations:** 1 UniCamillus-Saint Camillus International University of Health Sciences Rome Italy UniCamillus-Saint Camillus International University of Health Sciences, Rome, Italy.

Dear Editor,

I have read with extreme interest the following publication by França et al.,^(1)^ published in *Revista Brasileira de Ginecologia e Obstetrícia* in 2024.

Quite surprisingly, I found that data used seems to have been already published in a previous paper of 2022.^(2)^ Beside providing an explanation for the use of published material, the authors should also clarify the following methodological flaws that may prompt to question their results and conclusions:

1. The article reuses the exact same dataset from the P5 Trial (ReBec UTN: U1111-1164-2636), conducted in 17 centers between 2015 and 2019, yet presents the analysis as if it constituted new evidence. This is misleading and obscures the fact that the study is not independent.
2. The reported predictive performance (AUC 0.978 for preterm birth <34 weeks) is implausible. Such near-perfect discrimination is unprecedented in obstetric prediction models and strongly suggests severe overfitting, manipulation, or fabrication of data.
3. The so-called validation is invalid. Splitting the same randomized trial cohort into progesterone+pessary and progesterone-only groups does not constitute independent validation but instead recycles the same dataset to artificially inflate performance metrics.
4. Analytic thresholds (false-positive rates of 10% and 20%) were chosen retrospectively and optimized after viewing the data. With 31 variables examined without adjustment for multiplicity, the analysis reeks of post hoc cherry-picking and data dredging.
5. The results are irreconcilable with the original findings of the P5 Trial (Obstetrics and Gynecology, 2022).^(2)^ That RCT showed no significant difference in adverse neonatal outcomes (19.2% vs 20.9%, adjusted RR 0.88; 95% CI 0.69–1.12) and only a modest reduction in preterm birth <34 weeks (9.9% vs 13.9%, adjusted RR 0.66; 95% CI 0.47–0.93). Deriving an AUC of 0.978 from the same dataset is inconsistent with these modest results and raises serious questions about authenticity.
6. The absence of protocol pre-registration for this secondary analysis, lack of external validation, and failure to share the dataset or analysis code prevent verification of the extraordinary claims. Extraordinary results require extraordinary evidence, yet this article provides only opacity and implausible findings.
7. Taken together, these issues go beyond methodological weakness. They raise the possibility of questionable research practices and even scientific misconduct, calling into question the integrity of the article and its conclusions.
