# Supplementary material for: Comments on: A new screening of preterm birth in gestation with short cervix after pessary plus progesterone
Source: Rev Bras Ginecol Obstet. 2025 Nov 18;47:e-rbgo98. doi: 10.61622/rbgo/2025rbgo98 (PMC12671669; doi:10.61622/rbgo/2025rbgo98)
Supplement: Related texts

# Reply: Repply to: A new screening of preterm birth in gestation with short cervix after pessary plus progesterone

**Authors:** Marcelo Santucci França, Valter Lacerda de Andrade, Alan Roberto Hatanaka, Roberto Santos, Francisco Herlanio Costa Carvalho, Maria Laura Costa, Gabriela Ubeda Santucci França, Rosiane Mattar, Ben Willem Mol, Antonio Fernandes Moron, Rodolfo de Carvalho Pacagnella

**Affiliations:** 1 Universidade Federal de São Paulo Escola Paulista de Medicina São Paulo SP Brazil Escola Paulista de Medicina, Universidade Federal de São Paulo, São Paulo, SP, Brazil.; 2 Impacta Digital Academy Data Science São Paulo Brazil Impacta Digital Academy Data Science, São Paulo, Brazil.; 3 Universidade Federal do Ceará Children and Adolescents Health Department of Women Fortaleza CE Brazil Department of Women, Children and Adolescents Health, Universidade Federal do Ceará, Fortaleza, CE, Brazil.; 4 Universidade Estadual de Campinas School of Medical Sciences Department of Tocogynecology Campinas São Paulo Brazil Department of Tocogynecology, School of Medical Sciences, Universidade Estadual de Campinas, Campinas, São Paulo, Brazil.; 5 Monash University Department of Obstetrics and Gynaecology Victoria Australia Department of Obstetrics and Gynaecology, Monash University, Clayton, Victoria, Australia.

Dear Editor,

We appreciate Dr. Unfer's interest in our publication and the opportunity to clarify the points raised in his letter. Critical discussion is fundamental to scientific progress, especially in complex areas such as preterm birth prediction. However, some of the concerns appear to arise from a misinterpretation of our study.

Our article does not present a new randomized clinical trial nor provide definitive proof of efficacy. It is an exploratory post hoc analysis of a previously published trial, designed to test a predictive model. Its purpose was to generate hypotheses and identify women at higher risk of prematurity, even when receiving preventive treatment. From introduction to conclusion, we explicitly state that our findings *may* assist in risk identification but still require prospective validation before any clinical use.^(1)^

It is well established that a considerable proportion of women experience preterm birth despite prophylactic measures such as progesterone, pessary, or cerclage. These refractory cases illustrate the limitations of current preventive therapies and the need for complementary approaches to identify, early in pregnancy, those most vulnerable to adverse outcomes. Recognizing this subgroup is clinically important: it enables closer monitoring, guides counseling and shared decision-making, and defines priorities for future research targeting women at highest risk.

This motivation underlies the analysis presented in *"A new screening of preterm birth in gestation with short cervix after pessary plus progesterone"*.^(1)^ The study used data from the P5 Trial,^(2)^ which compared pessary + progesterone versus progesterone alone in women with short cervix. As a secondary *post hoc* analysis, it sought not to test intervention efficacy but to explore predictors of poor prognosis. This nature exempts the analysis from preregistration. The article clearly states that it is a secondary analysis with a distinct aim, and there was no attempt to conceal this.

The original P5 Trial showed a modest reduction in preterm birth <34 weeks and no significant difference in composite neonatal outcomes. Our analysis does not contradict these results. The high AUC reported does not reflect superior clinical efficacy of pessary use but rather model fitting within a homogeneous population of women with short cervix, in which categorical variable vectorization likely enhanced discrimination. Such performance, while interesting, must be interpreted cautiously because, as highlighted by Dr. Unfer, it may reflect overfitting.

The study, however, adds incremental information to the field. It reinforces that prematurity is a multifactorial condition that cannot be predicted by cervical length alone. Combining demographic, obstetric, and morphological variables improved risk discrimination. The model identified previous preterm birth, early detection of short cervix (<19 weeks), very short cervix (≤14.7 mm), and white ethnicity as main risk factors. Protective factors included no prior curettage, singleton gestation, and curved cervical length >21 mm, findings consistent with established biological plausibility regarding cervical integrity, obstetric history, and anatomic configuration.

These parameters merit consideration in future screening strategies so that more vulnerable women can be recognized early and followed closely. Nevertheless, the algorithm described should not be used universally. Predictive variables identified in one cohort may not hold in others, since cervical length distribution, ethnicity, and environmental influences vary among populations.

Validation in our study constitutes derived internal validation using two approaches: comparison with cervical length <15 mm and application of model coefficients to the progesterone-only group. This derived internal validation is not equivalent to independent external validation. The drop in model performance in the progesterone-only group demonstrates that accuracy decreases in broader or more heterogeneous samples. Precisely why extrapolation to clinical practice is premature and could lead to bias or misinterpretation. Prospective multicenter validation across diverse settings is essential to confirm reproducibility and ensure that apparent performance is not driven by homogeneity of the original cohort.

Regarding methodology, we used fixed false-positive rates (FPR 10% and 20%), following established precedents in obstetric screening.^(3-5)^ This approach allows balanced comparison of detection rates and reflects international standards for predictive modeling. Fixed FPRs are common in prenatal screening because they balance identification of high-risk patients with avoidance of excessive false alarms that may prompt unnecessary procedures.

On the matter of data and code sharing, we acknowledge this as a limitation of many papers, but this does not constitute misconduct. We support initiatives such as TRIPOD-AI^(6)^ that promote responsible transparency and reproducibility and agree that progress will depend on institutional and editorial policies that encourage data sharing without compromising the ethical protection of patients or reinforcing a colonialist stance on scientific data.

It is important to reaffirm that *post hoc* analyses such as ours are not intended to guide immediate clinical decisions or to establish definitive evidence. Their purpose is to generate hypotheses and stimulate scientific discussion. While methodological criticisms about overfitting and limited validation are legitimate, allegations of data manipulation or fabrication are unfounded. Our analysis represents a statistical exploration with preliminary findings, not a re-evaluation of treatment efficacy. The results should therefore be viewed as hypothesis-generating, pending confirmation in prospectively designed independent cohorts.

In conclusion, the model proposed is not intended for immediate clinical implementation, but it reinforces the multifactorial nature of the condition and demonstrates that multivariate modeling may help identify high-risk women even among those receiving prophylaxis. The appropriate next step is to externally validate the model, conduct prospective, multicenter studies including more diverse populations and determine whether recognition of this subgroup enables tailored interventions capable of reducing preterm birth. Only after such rigorous evaluation should broader clinical use be considered.

## References

[1] França MS, de Andrade VL (2024). A new screening of preterm birth in gestation with short cervix after pessary plus progesterone. Rev Bras Ginecol Obstet.

[2] Pacagnella RC, Silva TV, Cecatti JG, Passini R, Fanton TF, Borovac-Pinheiro A (2022). Pessary plus progesterone to prevent preterm birth in women with short cervixes: a randomized controlled trial. Obstet Gynecol.

[3] Mercer BM, Goldenberg RL, Das A, Moawad AH, Iams JD, Meis PJ (1996). The preterm prediction study: a clinical risk assessment system. Am J Obstet Gynecol.

[4] Chiu CP, Feng Q, Chaemsaithong P, Sahota DS, Lau YY, Yeung YK (2022). Prediction of spontaneous preterm birth and preterm prelabor rupture of membranes using maternal factors, obstetric history and biomarkers of placental function at 11–13 weeks. Ultrasound Obstet Gynecol.

[5] Gudicha DW, Romero R, Kabiri D, Hernandez-Andrade E, Pacora P, Erez O (2021). Personalized assessment of cervical length improves prediction of spontaneous preterm birth: a standard and a percentile calculator. Am J Obstet Gynecol.

[6] Collins GS, Moons KG, Dhiman P, Riley RD, Beam AL, Van Calster B (2024). TRIPOD+AI statement: updated guidance for reporting clinical prediction models that use regression or machine learning methods. BMJ.
